# Effects of virtual learning environments: A scoping review of literature

**DOI:** 10.1007/s10639-021-10768-w

**Published:** 2021-10-06

**Authors:** Laura Caprara, Cataldo Caprara

**Affiliations:** Graduate Studies in Education, St. Francis Xavier University, Antigonish, Nova Scotia Canada

**Keywords:** Virtual, Education, Learning, Mental health, Face-to-face, Synchronous

## Abstract

The purpose of this scoping review is to isolate and investigate the existing data and research that identifies if the synchronous face-to-face visual presence of a teacher in a virtual learning environment (VLE) is a significant factor in a student’s ability to maintain good mental health. While the present research on this explicit interaction among VLE implementation and student mental health is limited, the material suggests a framework for strong utilization of VLEs. Overall, our research has shown that authentic, high quality VLEs are ones that have as their primary focus the communication between students and their teachers and between students and their peers. This communication is best generated through synchronous connections where there exists the ability to convey the student’s immediate needs in real-time. Our research results and discussion will outline how a team approach that brings together teachers, students, administration, counsellors, mental health support staff, instructional designers, and ICT specialists is necessary to create a genuinely enriching VLE where both learning and social-emotional needs can be met. The authors present a case for further study in order to reveal the nature of the interaction among VLEs and student mental health.

## Introduction

Increasingly educators are turning to digital online tools and resources to supplement and enhance their teaching in the classroom. Independent of the move to online learning as a result of the COVID-19 pandemic, there is a clear upward trend to the prevalence and variety of online learning opportunities available to students of all ages. In their meta-analysis, Means et al. (2009) show that the rise of online learning is for a variety of reasons:

Online learning has become popular because of its potential for providing more flexible access to content and instruction at any time, from any place. Frequently, the focus entails (a) increasing the availability of learning experiences for learners who cannot or choose not to attend traditional face-to-face offerings, (b) assembling and disseminating instructional content more cost efficiently, or (c) enabling instructors to handle more students while maintaining learning outcome quality that is equivalent to that of comparable face-to-face instruction. (p. 22)

In Ontario, online learning can be accessed through traditional school boards using eLearning Ontario and Brightspace or through available online private schools such as VirtualHighSchool.com. These virtual learning environments (VLEs), online spaces that facilitate the delivery of curriculum content, assessment, and evaluation activities – deliver said curriculum in an asynchronous manner, that is “learning that is not delivered in real time. Asynchronous learning may involve students watching pre-recorded video lessons, completing assigned tasks, or contributing to online discussion boards” (Ontario Ministry of Education, 2020, PPM 164). This is different than synchronous learning which is defined as “Learning that happens in real time. Synchronous learning involves using text, video, or voice communication in a way that enables educators…to instruct and connect with students in real time” (Ontario Ministry of Education, 2020, PPM 164). It is believed that “synchronous learning supports the well-being and academic achievement of all students…by providing educators and students with an interactive and engaging way to learn. It helps teachers provide immediate feedback to students and enables students to interact with one another” (Ontario Ministry of Education, 2020, PPM 164). Despite this fulsome definition of synchronous learning, little research-driven policy or infrastructure exists whereby educators can be trained and supported in creating an ideal VLE for their students’ educational and social-emotional needs (Jones, 2015; Kent et al., 2018). This was certainly the case for all Ontario educators when, in March 2020, schools were closed due to the SARS-COVID-19 global pandemic and learning shifted to online-only instruction as contextualised in the following section.

### Background

On Thursday, March 12, 2020 at 4:00p.m. we tuned into the local news channel to hear that the Ontario Provincial Government was putting the province into lockdown to avert the upcoming threat of the novel Coronavirus, SARS-COVID-19. In this same news conference, we learned that Friday, March 13, 2020 would be our last day in schools; schools would be closed for two weeks after the March Break which began the following Monday. We spent the day photocopying, distributing, and explaining work packages for our students while ourselves wondering how we would negotiate the transition to distance learning and the new expectation to work – teach – from home. After these two weeks, a continuation of the lockdown was announced and Ontario educators were tasked with connecting with students and families procedure to gauge student’s communications technology readiness, to monitor their social-emotional needs arising from the lockdown and to explain that basic emergency learning was now in place.

At the same time, educators were told to create virtual learning environments (VLEs) to deliver this emergency instruction. Hours of instruction guidelines were provided by the Ontario Ministry of Education (April 2020): for JK-Grade 6, no more than six hours per week; for Grades 7–8, no more than eleven hours per week and for Grades 9–12, no more than three hours per course per week. During this time, normal attendance procedures were paused and replaced with anecdotal teacher monitoring and the mode and delivery of teaching was not standardized via a specific virtual platform. Guidance from Ontario teacher unions was to avoid videoconferencing and webcasting (OECTA, 2020) to maintain the privacy of both teacher and student.

The unprecedented scope and unforeseen turmoil caused by the COVID-19 crisis resulted in a delayed and mixed response for how educators could meet the daily social-emotional needs of their students who were now being taught in ways that were not conducive to learning such as having no face-to-face communication. Part of this mixed response included the ministry directive that students’ marks and grades would be frozen to the date prior to the initial closure (Miller, 2020), though opportunities for improvement would be provided. While the intentions of policymakers were rooted in compassion and wishing not to amplify any feelings of stress, anxiety, or hardship already being caused by the pandemic, it did not serve to inspire students to be continually engaged in their educational community or to feel wholly supported by said community. Overwhelmingly, students in both of our schools stopped engaging in course content; knowing that their grades could not change truly impacted their motivation, diligence, and general commitment to learning. When this disconnect occurred, teachers were advised to continue to call the student and encourage them to login to their VLEs, but this consistent check-in by four teachers at least once a week to each family began to feel at best like nagging and, at worst, an intrusion. This disconnect had another negative effect; now that students were not explicitly required to complete work for school, businesses were free to demand that they work throughout the day. Many students shared that they had taken on full-time hours at their part-time jobs. Had the student–teacher-classroom relationship continued throughout the closure somehow through routine, synchronous, live-video or live-voice communication, perhaps students and families would have felt a social and emotional pull to preserve their work habits and would have benefitted from helping to maintain the class community.

Essentially, our students went from daily, face-to-face interaction that built relationships and promoted positive social-emotional skills to the bare minimum interaction of a posting or an email a few times a week. Compounding this growing detachment was the social isolation that being in lockdown necessitated both from friends and family members who were at work. Students were at home, cut off from teachers, school, and friends, alone with the computer screens.

When schools reopened toward the end of September 2020, protocols and safety standards had been improved and implemented in ways that would encourage some families to opt for in-person learning while for others, the risk was still too great. For these families, school boards in Ontario created whole virtual schools or adopted a form of hybrid learning that would accommodate student from Junior Kindergarten through to Grade 12.

Our personal experience of in-person learning after the initial lockdown was atypical: though there was slight excitement the first day, this waned quickly and by the end of the week students were behaving very differently than they had been in March. Classes were silent; students were not speaking to each other, and group discussion or student–teacher conferences were very difficult to craft and nurture. In cases where students were priorly familiar with one another and the teacher, as was the case in one author’s classroom, students remained quiet, distanced, and generally withdrawn. These personal observations were similarly attested by several colleagues. For one author who had been assigned to the new virtual school, similar observations were made. Here, students from all over the city were being placed in VLEs together, disconnected from their home schools. Every attempt was made to create positive community bonds but still, save a handful of eager students, online classrooms were quiet both auditorily and in the chat box.

Unfortunately, COVID-19 continued to spread rapidly through the province and by the winter of 2021, the threat of the new, more infectious variants of COVID-19 forced the hand of the Ontario Ministry of Education to close schools once again for the remainder of the school year. The most marked difference in this lockdown is the continuation of the collection of attendance – students are at least driven to login to their VLEs to have their attendance recorded to avoid the consequences of truancy. Many students have shared with the authors that this second lockdown had evaporated their hopes of any social-emotional normalcy for their educational experience. They have attested that they are tired of feeling alone, overly challenged at the thought of having to continue their learning experiences solely through their computer screen.

## The path to inquiry

In the summer of 2020, The Hospital for Sick Children (2019) released a report that detailed how online learning and increased screen time could result in an increase of negative mental health effects. In addition, the prevailing advice of Public Health Ontario (2015) for the total number of hours of screen-time children and youth should be engaged in had not moved from the recommended “no more than 2 hours per day”. Instead of heeding the warning and advice of these public institutions, the Ontario Ministry of Education Policy/Program Memorandum No. 164 (2020) defining synchronous learning as “using text, video, or voice communication” in real time, set in place the following expectations for daily synchronous learning: 180 min for Kindergarten and 225 min for Grades 1–12.

Our desire to study the mental health effects of online learning takes its root in the experience of students and their guardians who have directly voiced difficulty with engagement in online schooling. Specifically, our students shared that their experiences in courses facilitated by teachers who did not utilize real-time video for the delivery of their synchronous lessons were difficult, hard to manage, and at times felt alienating.

Certainly, the few noted anecdotes above cannot constitute a direct cause for this review. Nonetheless, it is the view of the authors that such comments appearing from within the unique context of the mandated and sweeping switch to online learning warrant an investigation of what research might exist to establish acceptable standards of VLE implementation. In the case of the COVID-19 context, it must be understood that the changes to educational architectures were motivated by the physical requirement of stopping the spread of disease rather than what research has shown to be the best mode of education for the learner and their mental health needs. Further, even within the confines of mass VLE implementation, deeming the use of real-time video as strictly optional instead of prescribing a definitive modality for engaging in best practice displays a gap in an understanding of VLE significance. Further still, as online learning continues to be a the only viable option for many families, educators in Ontario must ensure that the creation of a VLE and the curriculum delivered through it is guided by the Ontario College of Teachers (OCT) *Ethical Standards of Care*, where it is understood that Ontario Teachers ought to avoid practices that may not support the welfare of all students placed in their care.

The Ontario College of Teachers (2020) defines the ethical standard of care as follows: “Members express their commitment to students' well-being and learning through positive influence, professional judgment and empathy in practice.” Further, the *Ontario Education Act, *1990 Sect. 264(1c) clarifies that part of a teacher’s duty is to impress the “highest regard for…humanity…and all other virtues” to their students. In addition to the duty of a Principal to provide “assiduous attention to the health and comfort of the pupils” (Ontario Education Act, 1990, Sect. 265(1j)) they must do so in such a way that they set the standard for the teacher. Without daily, live interaction with students, how is it possible to wholly meet these expectations?

Interestingly, the concept of all persons having to remain at home during the new COVID-19 online education paradigm seemed to create a vague understanding of whether it was even possible for the professional liability of care to take place. In consideration of the area of attendance, it must be understood that the traditional taking of attendance has a layered purpose: to ensure the “safe arrival” of students, to give proof of student attendance for funding allocation, and to link a student to the legal liability of a teacher as well as to allow teachers to exercise interventions for students who are truant. In the first stage of lockdown, teachers were asked to not take attendance and so were unable to ensure regular student contact. In this way, it became much more difficult to distinguish which students were not able to keep up with their studies and who required appropriate interventions for their learning, development, and wellness.

Tronick et al. (1978) showed that when the face-to-face interaction between infants and their mothers becomes distorted in such a way whereby the mother is unresponsive and still, “infants reacted with intense wariness and eventual withdrawal” (p. 1). They concluded that it was vital to have “interactional reciprocity” (Tronick et al., 1978, p. 1) to learn how to regulate their own emotional reactions. In their meta-analysis of this Still-Face Paradigm, Mesman et al. (2009) found that not only had the paradigm been consistent through multiple studies in infants, but its negative effects could also be found in both youth and adults. Experiments with adults resulted in “quite severe disrupting effects on social interactions, making people angry, confused, or upset…[where] [t]he perceived necessity for following the ground rules of social interactions is likely to stem from the evolutionary roots of human social life” (Mesman et al., 2009, p. 156). These results would suggest that part of the educator’s duty of care to a student is to maintain this quintessentially human behaviour of consistent face-to-face interaction. Without this fundamental interaction, it seems that humans are unable to properly regulate their emotional expression. Certainly social-emotional learning and the formation and maintenance of positive relationships with others is a core part of the *care* mandate of teachers.

## Purpose of scoping review

While it could be surmised that the guiding education authorities’ combined lack of clarity in declaring best practice directives and providing systems of care contributed to negative student experiences, the notion of how these organizational failures might have impacted student mental health must be drawn into focus. Indeed, the most notable shift in education has been toward the use of the online classroom and it is foreseeable that the continuation of this paradigm will be unavoidable. However, with the ambiguous messaging that surrounds that which defines quality implementation of a VLE, there lies a discernible gap in gauging the importance of teachers using live video in the delivery of synchronous instruction and if significant mental health implications are present in the decision to do so.

The purpose of our scoping review is to isolate and investigate the existing data and research that identifies if the real-time visual presence of a teacher in a VLE is a significant factor in a student’s ability to maintain good mental health. Such research might reconcile the void of definitive directives educational authorities have offered concerning teachers’ utilization of real-time video in a VLE and, more importantly, provide students with a higher standard of care while enduring the context of increased vulnerability the pandemic has introduced.

In short, this scoping review aims to answer two questions:

1. Does the synchronous face-to-face and interactive presence of a teacher in a VLE contribute positively to student learning and mental health and well-being?
2. What are the characteristics of a VLE that meets the social-emotional care requirements and needs of the student?

## Method and parameters

This scoping review works to capture the current “size and location of the literature” (Anderson et al., 2008, p.7) to better define the state of understanding of our research questions. Further, it is our hope that this scoping review might work to ascertain common threads among the research and note any gaps to inspire further inquiry in the topic. All of the material will be charted so it can be viewed in tandem for the above purposes.

### Boundaries of the review

Our search was limited to English-only peer-reviewed research literature that investigated the efficacy of online teaching and VLEs for educating the whole student. More specifically, we completed a search of all research articles published between 1 January 2010 and 31 January 2021 (an 11-year period). The rationale for focusing on this span of just over a decade was to draw our attention to the most recent research evidence related to our question. This date range also reflects the time span in which we had secured permanent work as educators; tracing the development and evolution of online learning since that point is of great personal interest.

Our search utilized the following terms: *virtual learning environment* (in all text) OR *online instruction* (in all text) AND *mental health* (in all text). These search terms were selected as they provided the widest catchment for the screening process required for this scoping review; in particular, the search term *virtual learning environment* was selected to identify research that was specific to an interactive context of online learning, as opposed to, for instance, online learning structured as a correspondence course. Databases that we utilized include ProQuest, ProQuest/ERIC, APA PsychINFO, and SAGE Journals Online because together they encompass a comprehensive and diverse catalogue of education-related literature as well as research regarding the psychological aspects of education. These database searches occurred between January 31, 2021, and February 7, 2021.

#### Special considerations of the topic search: defining a student

Ideally, the search terms could have been narrowed to refer to students specifically in a high school setting, using common platforms such as Google Classroom, but these terms did not yield any results. In an effort to compile the most useful sources for our research, a broadening rather than narrowing approach to our search terms was employed as it was discovered that limited resources existed in the field we were exploring. Considering this, the limits we set required special considerations of demarcation. For instance, many sources pertaining to the student mental health in a VLE context dealt exclusively with nursing students (e.g., Shea & Rovera, 2021). These studies offered excellent data sets but were omitted because the data could not be understood as congruous with a universalized definition of what constitutes a student. That is, the specialized nature of these studies involved students who had the responsibility of dealing with patient care, using VLEs to interact with their patients. In this way, the nursing paradigm muddied the demarcation between the VLE experience of a student and that of an authority.

### Inclusion criteria

In initially assessing and screening the results of the database searches, we ventured to remove sources that did not pertain to the contexts of VLEs and the student’s experience of those VLEs. Here, a primary list of 268 articles was redacted to a sum of 63 articles via an evaluation on the content of the title and abstract of each article. The inclusion of the remaining 63 articles was based on their titles and abstracts having expressed: (1) the use of an online modality that would implicitly involve the visual presence of an educator, and (2) the interest in the contexts of the learners’ experiences being a result of the instruction provided. Sources that were concerned with the experience of post-secondary students were only included if they involved Year 1 students as part of their purview. This limit was imposed on the research as we perceived this domain of data as relevant to the prospect of constructing an information-set surrounding the transitional stage of Grade 12 into post-secondary. Further, this inclusion might allow for a greater basis of insight into the still emerging reality of widespread online education. Notable exclusions from the research that might have helped develop such a picture were many articles that focused on the VLE experiences of graduate and post-graduate level students (e.g., Chugani & Houtrow, 2020; Gardner, 2020; Shawaqfeh et al., 2020) and articles concerned with the mental health of educators who utilized VLEs (e.g., Watermeyer et al., 2021; Rowe et al., 2020; Ault et al., 2020; Alkarani & Thobaity, 2020; Schlesselman, 2020). The subsequent 63 articles were read in their entirety to determine whether their content would be appropriate for this scoping review. This stage of assessment resulted in the inclusion of 38 articles.

### Manual inclusions

**Fig. 1:**
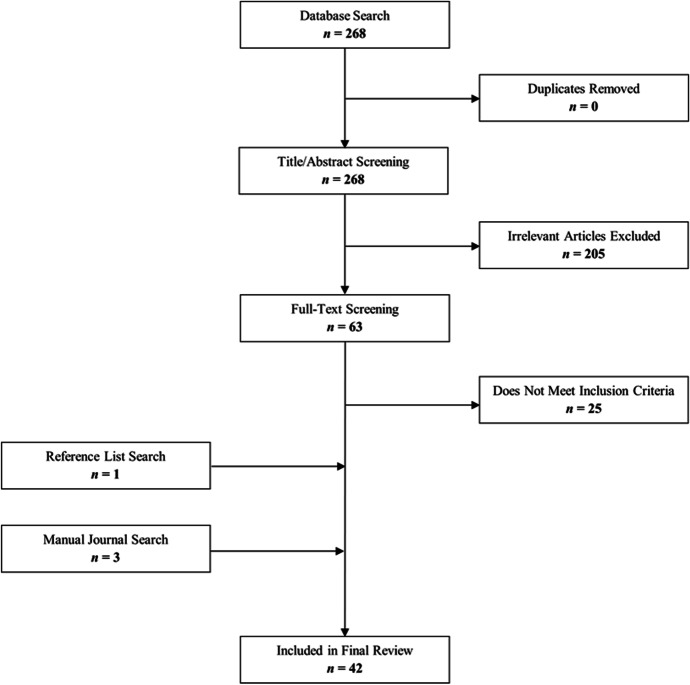
Review process and results

Additionally, a reference check of all 38 articles yielded the inclusion of a meta-analysis authored by Cavanaugh et al. (2004) we deemed relevant insofar as it provided an overview of the effects of online learning in a strictly academic sense allowing a possible comparison between achievement and positive mental health. A manual search conducted prior to the exercise of prescribed database search-terms warranted the inclusion of one other meta-analysis authored by Mesman et al. (2009) which is noted in the introduction section of this scoping review. The specific data and information presented in the work of Mesman et al. (2009) serves as a correlational tool in observing the trends apparent in the included articles. As a final article addition, a second manual journal search was conducted where two studies were found to include data that encompassed student perceptions of their experiences with VLEs, thus, resulting in a total sum of 42 articles to be included in this scoping review. The total review process is noted visually in Fig. 1.

## Summary of research findings

The purpose of this scoping review is to isolate and investigate the existing data and research that identifies if the real-time visual presence of a teacher in a virtual learning environment (VLE) is a significant factor in a student’s ability to maintain good mental health. Overall, our research has shown that authentic, high quality VLEs are ones that have as their primary focus the communication between students and their teachers and between students and their peers. This communication is best generated through synchronous connections where there exists the ability to convey the student’s immediate needs in real-time. The demands placed upon an individual educator to facilitate an online education for students, inclusive of the creation and maintenance of an effectual and engaging VLE often serving as a proxy to a face-to-face (F2F) base-school, “require[s] extensive time commitments” (Wingo et al., 2016, p. 437) which are far beyond the standard workload formulas calculated by many institutions (Wingo et al., 2016). Indeed, the complexity of this task while also providing sufficient social-emotional services for students and families is not fully understood in terms of student and teacher equity. Our research results and discussion will outline how a team approach that brings together teachers, students, administration, counsellors, mental health support staff, instructional designers, and ICT specialists is necessary to create a genuinely enriching VLE where both learning and social-emotional needs can be met.

Summaries of the 42 articles pertaining to this research are provided in Table 1. In addition to information related to authors, years of publication, countries (i.e., where the research was conducted), participant profiles, intervention programs and timelines (where available) and data sources, we have also summarised the researchers’ aims, research designs, findings, and conclusions.

**Table 1:** Research summary of the various iterations of virtual learning environments (VLE)

Author(s), Year, Country	Aim (A), Research Design (RD), and Intervention Program (IP), if any	Participant Profile (number of students; age; control (CG) and intervention groups (IG), if applicable)	Data Source(s) (DS) and Timeline of Data Collection (TDC), if applicable	Statistical Significance (if reported or applicable), Findings, and Conclusions
Archambault et al., 2013, U.S.A	A: To examine how truancy laws can and do apply to online students, and (2) to determine the responsibility of online schools to enforce state attendance laws. RD: Case-study design	Policies at Minnesota Virtual High School (MVHS) that educates approximately 1350 students, grades 6–12; Dean of Students at MVHS	DS: Minnesota Attendance and Truancy Statutes; Chicago Country Truancy Policy; Structured interview transcript	(1) Indeed, online schools are public schools, and its students are subject to state attendance and truancy laws. (2) MVHS uses a competency-based calculation to determine attendance; work completion and skill progress are calculated as percentages that correspond to the number of school days per week. Virtual schools need to take an active role in overseeing students’ progress, which may require additional staff resources.
Baker et al., 2019, U.S.A	A: To test the efficacy of a scheduling intervention in an online post-secondary course. RD: Experimental IP: Scheduling the watching of the lecture	*N* = 145 Undergraduate students mean age = 20 yrs (SD = 1.2)	DS: Pre-intervention survey; Weekly quiz scores; Clickstream data from VLE TDC: 2 weeks of a 6-week course	This intervention is most helpful for students who believe that their time management skills are poor, regardless of their actual skills. Effects diminish over time, however.
Barbour & LaBonte, 2019, Canada	A: (1) To examine the existing system of e-learning in Ontario, (2) to investigate the 2019 mandatory e-learning proposal, and (3) to investigate lack of research to support the proposal. RD: Literature Review	87 documents	DS: the findings of 87 articles consisting of institutional research studies, governmental policies, and institutional surveys	(1) Centralized vs. decentralized delivery is insufficient; both offer outcomes that are similar to F2F contexts. (2) No evidence to show that e-learning creates a negative impact on graduation rates. (3) Connection between increases of e-learning class size and negative impact on student achievement outcomes.
Blayone et al., 2018, Georgia & Ukraine	A: To determine the state of digital readiness of Georgian and Ukrainian students for online learning over 4 variables: technical actions, communication, informational, and computational. RD: Quasi-experimental	*N* = 279 Georgian students, *n* = 150; Ukrainian students, *n* = 129 Age range of 17–30	DS: Survey using the Digital Competency Profiler (DCP) survey	In both cohorts, (i) mobile device usage signified the highest readiness, chiefly with word-processing; (ii) avid use of mobile devices for social media did not translate to high readiness for e-learning communication; (iii) low readiness for searching for research texts; (iv) up to 96% have low readiness if using mobile devices for computation. Technology readiness is needed but is not a sufficient condition for building successful e-learning experiences.
Cairns et al., 2020, U.S.A	A: To provide academic insight into the variety of experiences and technological platforms used during crisis remote learning. RD: Qualitative, exploratory	*N* = 28 Undergraduate students, Years 1–4	DS: Interviews	Technology facilitates connections, new modes of interaction, and meaningful relationship building. There is a hierarchy in connection preferences, with in-person first, then video, then talk, then text and in those modalities, synchronous connection is preferred over asynchronous. As well, F2F or video connection is deemed more meaningful for its ability to show facial expressions.
Cavanaugh et al., 2004, U.S.A	A: (1) To collect data and draw conclusions on student learning in online programs and how it compares to learning in classroom-based programs, and (2) to identify the specific factors that influence student learning. RD: Meta-analysis	Based on 116 effect sizes from 14 web-delivered, grades 3–12 distance education programs: combined sample size of *N* = 7561 students	DS: The findings of 14 studies	The mean effect size across all outcomes (*r* = -0.028) indicates no significant difference in performance of online vs. F2F class. The unweighted effect sizes (*r* = -1.158 to 0.597, SD = 0.157) indicate that some applications of distance education appeared to be much better than F2F and others were much worse. Distance education can have the same effect on measures of student academic achievement when compared to traditional instruction. They can be seen as ‘equivalent’.
Crea & Sparnon, 2017, Kenya and Jordan	A: (1) To review the implementation of an online program that provided tertiary education to refugees and, (2) to explore the benefits and drawbacks of the program. RD: Qualitative IP: Diploma Program created for students in refugee camps consisting of 15, 8-week courses over 3 years. On-site staff would care for the physical, legal, and social-emotional needs of students, while international faculty would instruct courses online off-site	*N* = 79 Refugee camp staff (‘on-site’), *n* = 23; international faculty (‘off-site’), *n* = 56	DS: surveys TDC: Every 8 weeks, feedback was solicited from students through staff/faculty; August 2013-March 2014	Faculty noted the importance of mentors working with students and the ability to communicate and collaborate with on-site members. Students report improved functioning in their communities and being better able to attain robust employment because of the program. Students also reported renewed sense of hope in their future due to online education. Despite fluidity of online option, the gender barrier of accessing education was too great to overcome for many women.
Davies, 2014, United Kingdom	A: To investigate the efficacy of using the iPad to facilitate student interaction, group cohesion, and the sharing of student material. RD: Quasi-experimental IP: To use an iPad to keep track of tutorial lessons and discussions	*N* = 80 Undergraduate students, Year 3 CG: *n* = 56, F2F; not measured IG: *n* = 24	DS: Descriptive feedback, surveys TDC: Midpoint and cessation of intervention	Results indicated that 85% of participants reported a positive outcome of having the opportunity to revisit material online after instructional sessions. Mobile computing platforms may help students to engage more fully with learning activities and materials, and increase student confidence with peer presentation and feedback.
Domina et al., 2021, U.S.A	A: To investigate predictors of elementary school student engagement during the initial period of the COVID-19 pandemic remote learning. RD: Causal comparative method	*N* = 9741 Parents and guardians (providing data on approximately 42% of all elementary students)	DS: Survey TDC: Surveys administered between May 26 and July 1, 2020	Significantly, for households with less than 1 device per child score lower on measures of engagement (SD = 0.13 to 0.32); students with access to high-speed internet score higher (SD = 0.32) than students without on the measure of success in completing assignments. Each additional socio-emotional learning opportunity is associated with a SD = 0.1 improvement in student enjoyment of remote learning (*p* < 0.001) and in their ability to complete and submit online work (*p* < 0.001) as well as the frequency with which students log on to remote instruction (*p* < 0.01). Students whose families remained socially connected to other students’ families were more likely to be engaged. Those with no instances of connection reported less enjoyment (*p* = 0.05) and less success completing the school year (*p* = 0.01). Multiple forms of remote instruction helped engagement (*p* < 0.001); these multiple modes were associated with greater student enjoyment (*p* < 0.001) and more frequent login (*p* < 0.001).
Dommett et al., 2019, U.S.A	A: (1) To investigate perceptions of video-captured lectures, and (2) their usefulness for learning. RD: Qualitative analysis	*N* = 25 Faculty, *n* = 8; students, *n* = 17	DS: Focus group transcripts	Students reported that captured video of class lectures were efficient for revision, low cost, an adequate substitute when attendance not possible, helpful for ELLs, and valued for narratives that elaborate on lecture slides. Of note, knowing the recordings are available had a positive impact on feelings of well-being and reduced anxiety in students with disabilities. Faculty felt that the recordings should be used minimally lest they become reductive tool. They also reported the worry that recordings reinforce students’ perception of the lecture serving only as information transfer or stand-alone events rather than part of a whole program.
Driscoll et al., 2012, U.S.A	A: To investigate whether student performance and satisfaction intrinsically differ across online and F2F classroom settings, independent of student characteristics. RD: Quasi-experimental IP: An online course focused on interaction, clear organization, and structure with a focus on content over delivery method will be offered to the IG, and a traditional F2F course offered to the CG	*N* = 368 Undergraduate students, Year 1 CG: *n* = 198 IG: *n* = 170	DS: Surveys (Cronbach’s α = 0.865); Academic achievement scores TDC: Surveys administered pre- and post-intervention, academic scores recorded at midpoint (midterm exam) and a final project	While mean comparisons of the dependent variables show that F2F students performed better on both assessments, this result is a potential selection effect with GPA as a predictor variable. There is no significant difference between satisfaction among the two groups. Results support that online education can be equally effective when an online course is designed using appropriate pedagogy. Students who enjoy working with others and view interaction with their instructor as important to learning tended to be more satisfied with the course, independent of the type of classroom they are in.
Du et al., 2019, U.S.A	A: To examine six factors related to students’ self-efficacy beliefs in online groupwork: online groupwork interest, technology and media use, willingness to handle online groupwork challenge, leadership, trust relationship and, online groupwork self-efficacy. RD: Quantitative survey	*N* = 204 Graduate students; 70% are < 30 years Participants split into 61 groups over 3 years: average of 3.35 participants per group	DS: Survey TDC: Survey administered at end of semester; over Fall 2009 – Spring 2012	Online groupwork self-efficacy is significantly positively correlated with age, gender, and experience with online courses. Students’ willingness to handle challenge is positively related to online groupwork self-efficacy (*p* < 0.01); only this willingness can predict online group self-efficacy. The more students trust and support each other within the group, the more efficacious they are doing groupwork (*p* < 0.01). The more positive perceptions of the leadership in the group, the more efficacious they are doing online groupwork (*p* < 0.05).
Engelbertink et al., 2020, The Netherlands	A: (1) To examine the needs of students and teachers that could achieve an optimal blend between F2F and online elements of a course, and (2) to discover if persuasive technology is suitable for BLMs. RD: Qualitative participatory design approach	*N* = 17 Teachers, *n* = 4; ICT specialist, *n* = 3; educational experts, *n* = 2; social workers, *n* = 3; and students, *n* = 5 (mean age = 19 yrs)	DS: Meeting minutes; Surveys; Descriptive feedback TDC: 6 meetings (2 h each over 6 months)	(1) Both teacher and student could motivate students to do their homework F2F; persuasive technology can ensure this F2F-based motivation is maintained during asynchronous time. (2) Persuasive technology is suitable for a BLM when using elements of primary task, dialogue, and social support. Of note is the need to regulate dialogue supports such as rewards and praise so that they stimulate learning, rather than becoming the learning’s extrinsic value.
Gibson & Smith, 2018, United Kingdom	A: (1) to present skills needed by students to navigate their information journey through online learning, and (2) discuss how educators can support students’ acquisition and development of these skills. RD: Systematic review	30 Studies	DS: The findings and conclusions of 30 studies	Educators must equip learners with autonomy to navigate searches for information. For primary learners, focus must be on fostering enquiry-minded researchers. For secondary students, lessons in critical reading, writing, and critiques of sources are essential. These lessons must be precise, concise, and targeted. For learners in higher education, institutions can support their learning by refining obtuse LMSs and use new and innovative tools instead.
Gillis & Krull, 2020, U.S.A	A: (1) To analyze student’s perceptions of the emergency transition to online learning due to the COVID-19 pandemic, (2) to evaluate the usefulness of various online learning strategies, and (3) analyze barriers encountered. RD: Qualitative	*N* = 66 Undergraduate students, Year 1 IG: (1) *n* = 40; (2) *n* = 26	DS: Surveys with open-ended questions TDC: At onset of emergency learning; 2 weeks into emergency learning; 1 week after course completion	(1) Live Zoom discussions structured similarly as F2F classes were rated highly with 70% of students rating them with ‘high effectiveness’ whereas only 41% found them effective in other courses. Regarding small group video, 83% rated it as ‘very accessible’ (vs. 70% in other courses) and 76% rated it as ‘effective’ (vs. 33% for other courses). (2) The study underlines the importance of gathering feedback from students at multiple points to ensure the identification of barriers to accessibility and effectiveness. While Zoom rated highly for effectiveness, the qualities of enjoyment and accessibility rated on par with other asynchronous interventions. (3) Results show high levels of anxiety, distraction, and lack of motivation for all students, but are particular evident in students who come from underserved backgrounds.
Ho et al., 2016, Vietnam	A: To examine the effectiveness of a BLM for teacher education students learning the ‘hands-on-approach’ method. RD: Quasi-experimental; mixed methods (qualitative & quantitative) IP: BLM where students engage in online, self-paced learning and collaboration on a product after an introductory F2F lesson	*N* = 177 Undergraduate students CG: *n* = 60, F2F IG: *n* = 117, BLM	DS: Assignment achievement scores; Test scores; Reflective questionnaire TDC: Before and after intervention	Students in the IG showed a significantly higher level of knowledge and overall satisfaction (*p* = < 0.05) as well as a statistically significant difference in achievement mean scores with the BLM students scoring higher (*p* = < 0.05). However, feelings of self-efficacy with the ‘hands-on-approach’ were not significantly different between the CG and the IG though learners in the CG group felt they could improve their self-efficacy due to the practical nature of the course delivered F2F. Effective BLMs are successful due to the following factors: access, flexibility, cost effective, improving interaction, formation of network, and involving school leaders.
Huang et al., 2011, Taiwan	A: To investigate whether a BLM with weblog and RSS technology can mitigate the social barriers found in classroom settings, issues of time lag, and cognitive overload. RD: Quasi-experimental comparison group IP: To complete course objectives using weblogs with jigsaw cooperative learning activities with RSS feeds	*N* = 115 Undergraduate students, Year 2 CG: *n* = 58, F2F IG: *n* = 57, online	DS: Questionnaires TDC: Questionnaires administered to all students at beginning of course; second questionnaire administered to only the IG	The IG had more positive impression of the jigsaw activity (*t*(1113) = 2.62, *p* < 0.01), felt less time pressure (*t*(1113) = 4.95, *p* < 0.01), and felt less peer pressure (*t*(1113) = 2.58, *p* < 0.05). Of the IG, 85% reported that the RSS with keyword search functions decreased cognitive overload and the time lag of information delivery while 60% indicated that the tag function was of neutral quality. Students must be taught to use these features of the IP to see maximal benefits.
Huang et al., 2019, U.S.A	A: To examine whether 3DVR technology contexts satisfies or obstructs psychological need and whether it fosters or undermines sustained engagement and behaviour. RD: Experimental IP: Students participate in a 3DVR Second Life simulation. A virtual tourism destination was developed with the learning objective to explore and be aware of Maasai Mara culture	*N* = 198 mean age range, 18–24 yrs CG: students experienced with 3DVR IG: students not experienced with 3DVR	DS: Self-administered questionnaire with closed-end questions; Observation TDC: Questionnaire administered at end of course; Observations taken during 3, 1-h classes for the CG and during 18, 2-h classes for the IG	Sense of relatedness predicted positive behaviour (*p* < 0.05) and had a positive impact on intrinsic motivation (*p* < 0.05); feeling effective predicted intrinsic motivation while in a VLE (*p* < 0.05); sense of autonomy in the VLE predicted positive behavioural intentions (*p* < 0.05) as well as positive emotions (*p* < 0.05). No significant relationship between perception of competence and intrinsic motivation (*p* > 0.05) or behavioural intention (*p* > 0.05).
Hursen, 2019, Cyprus	A: (1) To determine the effects of problem-based learning activities supported with Facebook on learner’s perceptions of self-efficacy for research-inquiry and, (2) to determine the views of students on problem-based learning in this context. RD: Mixed method study (qualitative & quantitative) IP: Using Facebook, students engaged in problem-solving activities	*N* = 25 mean age = 28 yrs	DS: Assessment test scores; Interviews TDC: Assessment was administered pre-IP and post-IP for whole group; interview was conducted post-IP for *n* = 14	While the dimensions of ‘avoidance’ and ‘personal development’ remained not significantly changed (pre and post), ‘sustained research’ showed significant positive change (*z* = -2.86, *p* < 0.05). Problem based learning activities supported by Facebook provided a positive increase in perceptions of self-efficacy for sustained research among adult students. This improvement is most notable in their specific self-efficacy perceptions for planning, focusing, and sustained research. The majority found the experience positive with the most notable negatives the result of Internet connectivity issues.
Jena, 2016, India	A: (1) To examine the inter-relation between student attitudes (SA), learning readiness (LR), and learning style (LS) in VLE-enabled higher education and, (2) to find any differences in attitudes based on students’ LS to establish whether a relationship exists between LR and LS in a VLE. RD: Experimental	*N* = 240 mean age = 22.8 yrs	DS: Questionnaires (Cronbach’s α = 0.90)	No significant differences of overall LS with respect to gender (*p* > 0.05) toward the use of VLE technology or toward the LR to use VLE technology (*p* > 0.05). The SA toward the use of VLE differ significantly with respect to their academic background (*p* < 0.05) but the LR of students toward the use of VLE does not differ significantly (*p* > 0.05) with respect to their academic background. The LS of students play a significant role in the prediction of student learning (*p* < 0.05).
Johnston et al., 2014, U.S.A	A: To examine 3 variables on the prevalence and quality of peer-to-peer (P2P) learning among students with disabilities: student aptitude for P2P learning, design of the VLE, and the social and pedagogical context where learning is targeted. RD: Comprehensive case study	*N* = 14: Teachers, *n* = 3; Administrators, *n* = 3; Data coaches, *n* = 2; caregivers, *n* = 3; students, *n* = 3 (mean age = 10 yrs)	DS: Individual, semi-structured interview transcripts; Focus group transcripts; Structured observation	A greater effort is required to satisfactorily support and train teachers and parents to explicitly identify and foster P2P learning to increase opportunities for all students to learn from each other while online. Without consistent P2P interaction, students with disabilities do not have the ability to grow in their independence and self-regulation skills.
Jones, 2015, U.S.A	A: (1) To describe the infrastructure and resources required for quality online learning, (2) to examine the benefits and challenges of teaching online vs. F2F, and (3) discuss the impact of the differences on students, educators, and practitioners. RD: Limited Case Study		DS: Author’s own experience of teaching online courses	(1) An office of distance learning is essential for establishing and maintaining standards of quality. (2) Benefits of teaching online include increased access and content that is comparable to F2F. Challenges of teaching online include lack of access to high-speed internet, extra tuition costs, less interaction and engagement, and extra time required. (3) Students and online teachers need training and equipment. More time is needed to adjust to the difference in the intense “presence” requirements. While writing online can allow for connection, only synchronous, real-time, video conferencing provided the necessary interpersonal interaction opportunities.
Kent et al., 2018, Australia	A: To report on the e-learning experience of those with disabilities, considering dimensions of accessibility and disclosure. RD: Correlational analysis	*N* = 125 mean age = 36 yrs	DS: Surveys with open response questions	Only 15% of enrolments are for the Humanities, but 44% of students with disabilities are enrolled in the Humanities. Of all students with disabilities, 48% did not receive accommodations for their disability; 24% experienced accessibility problems due to disability. The findings show that there is an increased need for universal design to reach all students, both in technology and pedagogy, especially considering that not all students disclosed their disability. There is also a need for disciplines outside of the humanities to reach out to students with disabilities.
Kumar & Owston, 2016, Canada	A: To determine the extent to which data from automated tools used to measure accessibility of e-learning units is predictive of the subjective experiences of students. RD: Case study	*N* = 24 Undergraduate students; Years 1–4 CG: automated tools (Achecker, Qompliance, WAVE, PPT, and Powertalk) IG: student users	DS: List of predicted accessibility barriers generated by automated tools; List of actual accessibility barriers generated by students; Post-experiment questionnaire; Semi-structured interview TDC: Midpoint and cessation of intervention	Student-centered methods are an essential component of e-learning accessibility evaluation. Automated tools and the accessibility conformance guidelines they are based on are not effective at identifying barriers to accessibility for e-learning and further, can omit barriers that may have significant impacts on students. More effort must be directed toward ensuring that students can understand the interface of the LMS.
Lan et al., 2018, Taiwan	A: (1) To establish the principles of designing a 3DVE for children with a disability for the purpose of learning their first language, and (2) to observe the effects of learning a first language in a 3DVE. RD: Design-based qualitative study IP: Using 3DVE Second Life simulation, students would engage with one another to practice their communication skills	*N* = 4 mean age range = 8–9 yrs	DS: Interview questionnaire with open response; Observations; Video recordings TDC: Daily in-class observations over months; Interview questionnaire administered at end of IP	For students with disabilities, difficulty in task should be gradually increased based on learner’s abilities. Voice functionality should be modulated as to not distract students and avatar and 3D item manipulation should be limited. Scene design should reflect student life experience. Interactive objects that can be manipulated interactively by multiple users are more popular with students. Children and their parents approved of learning by playing within the 3DVE as it increased their child’s linguistic communication skills.
Lee et al., 2016, U.S.A	A: To investigate whether online group work increased students’ satisfaction with interaction among students in an online course. RD: Quasi-experimental IP: Three identical online courses assigned the same project; CG will work individually, and IG will work in small groups online	*N* = 283 Undergraduate students, Year 1 CG: *n* = 94 IG: *n* = 189	DS: Survey with open-ended questions TDC: Survey administered 1 week after project due date	The group assignment did not influence students’ satisfaction (*p* = 0.407) with online interactions. Instead, students’ perceptions of the importance of interaction were associated with their satisfaction with interaction in the online course (*p* = 0.050). Additionally, the IG did not believe they learned more from the group than they would have if learning on their own. Online instructors and instructional designers need to do more to ensure LMS tools contribute to successful online collaboration.
Lee & Oh, 2017, South Korea	A: To examine the role of perceived stress on the relationship between academic stress and depressive symptoms among e-learning students with visual impairments. RD: Correlational analysis	*N* = 103 mean age = 41.3 yrs	DS: Perceived Stress Scale survey (*PSS-10*, Cronbach’s α = 0.816); Depressive symptoms survey using the Center for Epidemiologic Studies Scale (*CES-D*, Cronbach’s α = 0.906); Academic stress survey using the Maslach Burnout Inventory-Student Survey (*MBI-SS*, Cronbach’s α = 0.912)	Academic stress was positively correlated with perceived stress (*r* = 0.355, *p* = 0.0001) and depressive symptoms (*r* = 0.337, *p* = 0.0001). Perceived stress was positively associated with depressive symptoms (*r* = 0.637, *p* = 0.0001). The association between academic stress and depressive symptoms was fully mediated by perceived stress. E-learning students with disabilities will experience these more acutely and thus are at higher risk.
Mallya et al., 2019, India	A: To examine the role of internet self-efficacy (ISE) as an antecedent construct to the technology acceptance model (TAM). RD: Quantitative	*N* = 448 Undergraduate students *n* = 287; Graduate students, *n* = 161	DS: Structured questionnaire	ISE has a significant influence on the perceived ease of using (PEOU) the Internet. ISE has a positive influence on both PEOU (*p* < 0.001) and perceived usefulness (PU) (*p* < 0.001). PU (*p* < 0.001) and PEOU (*p* < 0.001) have a positive influence on attitude toward using the internet (ATI). ATI (*p* < 0.001) and PU (*p* < 0.001) have a positive influence on behavioral intention towards internet (BI) and intend to use it for academic activities. The higher the self-efficacy, the higher will be their intention to use the internet for learning purposes.
Manthey et al., 2016, Germany	A: To examine the effects of regularly practicing cognitive interventions on subjective well-being using online video tools. RD: Experimental IP: Using online video instructions, IG1 will engage in reflective exercise regarding their ‘best possible selves’; IG2 will engaged in reflective exercise making ‘gratitude lists’ and, the CG will create ‘to-do lists’	*N* = 435 Student volunteers, age range 18–63 yrs CG: *n* = 150 IG: (1) *n* = 135; (2) *n* = 150	DS: Written reflections TDC: 2-month intervention; 1 month follow-up	Findings revealed that both interventions significantly increased subjective well-being in comparison to the control condition and thus, instructional internet video-based interventions have a direct effect on subjective well-being. These effects can be sustained over a 1-month follow-up period and are especially effective when the person-intervention fit is high. The effect size among the pre- and post-interventions ranged from *r* = 0.09-0.13.
Merlin-Knoblich et al., 2019, U.S.A	A: To examine student engagement in flipped and non-flipped courses. RD: Causal comparative method IP: Two identical online classes except the IG cohort completed readings and work online prior to attending the F2F component which included practice activities, and the CG did all the work in the F2F sessions	*N* = 67 graduate students CG: *n* = 37 IG: *n* = 30	DS: Questionnaires TDC: Questionnaires administered at the end of a 15-week semester	The IG self-reported significantly more affective engagement than the CG (*p* = 0.013, *d* = 0.61); significantly more behavioural engagement in terms of compliance compared to the CG (*p* = 0.038, *d* = 0.50); significantly more cognitive engagement than the CG (*p* = 0.013, *d* = 0.64); and significantly higher perceptions of overall engagement than the CG (*p* = 0.005, *d* = 0.70).
Mesman et al., 2009, The Netherlands	A: (1) To provide a structured insight into the themes that have been addressed using the still-face paradigm (SFP) as well as clarify reasons for and consequences of variations in the basic SFP procedure through a systematic review, and (2) to conduct a meta-analysis of studies that have used the SFP standard procedure to understand the still-face effect (SFE), the subsequent recovery effect, and a potential carry-over effect in terms of their magnitude for different behaviours and of potential sample and procedural moderators of these effects. RD: Systematic narrative review and meta-analysis	85 Studies for narrative review 39 Studies for meta-analysis	DS: The findings and conclusions of 124 studies	(1) The systematic narrative review indicates that the SFE that occurs is due to the break in normal social interaction rather than boredom and this effect is found across different samples in terms of demographic and risk variables. (2) The meta-analysis confirmed the findings of the systematic review in many ways. There was a decreasing positive affect and gaze and an increasing negative and neutral affect from the baseline to the still-face episode. As well, the hypothesized carry-over effect was found for positive and negative affect, regardless of procedural variations. Significantly, the SFE is universal among Western societies, in infants of varying ages, both genders, and those with normative development. Where there is a higher maternal sensitivity and positive behaviour there is more positive affect and less avoidance/negative affect.
Papanastasiou et al., 2019, Greece	A: To explore a sample of representative studies affecting K-12, higher and tertiary education students that highlight the theoretical and practical aspects of the use of VR/AR technology. RD: Literature Review	39 studies	DS: The findings of 39 studies	VR/AR technology can improve learning outcomes and are advantageous in terms of time and financial investment in K-12, higher and tertiary educational settings. These tools improve digital-age literacy, creative thinking skills, communication, collaboration, and problem-solving ability. VR/AR also enhances positively traditional curricula, especially for learners with disabilities. This technology also raises student engagement, promotes independent learning, enhances multi-sensory learning, confidence, and overall enjoyment.
Pryjmachuk et al., 2012, United Kingdom	A: To explore whether an online study skills course was ‘fit for purpose’, and (2) to test its effectiveness. RD: Mixed methods, concurrent, evaluative design	*N* = 63 mean age = 28.5 yrs	DS: Surveys (*n* = 63), Interviews (*n* = 12) TDC: Surveys administered at beginning, mid-point, cessation, and the interviews at follow-up	Survey reveals increased confidence across all fields with before and after median knowledge scores being 58.3% and 70.8% respectively. An online study skills course unit, designed using evidence-based principles, can produce benefits for students, especially in terms of increased confidence.
Roblek et al., 2019, Slovenia	A: To present how contemporary students are self-organizing using smart technologies (ST), and (2) to explore the future social implications of ST. RD: Narrative analysis and interpretation of qualitative data	*N* = 144 Undergraduate students, Years 1–4	DS: Questionnaire; Personal reflections	Students mostly use ST to save time while studying and for leisure. ST is so intertwined with their studies, work, and spare time that students can no longer conceive of their lives without it. Despite this deep integration of ST, students are concerned with issues of privacy and the effect ST will have on spending time with loved ones.
Rogowska et al., 2020, Poland	A: To examine the association of anxiety with self-rated general health, satisfaction with life, stress, and coping strategies of University students during the COVID-19 pandemic outbreak in Poland. RD: Qualitative	*N* = 914 Undergraduate students; median age = 23 yrs	DS: Standard psychological questionnaire	Overall, 65% of students suffered from generalized anxiety disorder (32% mild; 21% moderate; 14% severe). Additionally, 56% reported a high level of perceived stress, shown to be a significant and positive predictor of anxiety disorders. Perceived stress, current health status, and general anxiety disorder correlate with each other at a high rate of statistical significance (*p* < 0.001). University students experience very high anxiety and stress during the COVID-19 pandemic.
Stone, 2019, Australia	A: To compare student and faculty perspectives on ways to improve outcomes in online learning. RD: Qualitative-correlational; Literature review	3 Studies (student total, *n* = 144; faculty total, *n* = 151)	DS: The findings of 3 research projects, which themselves incl. surveys and interviews	Several issues were shared by both groups: lack of inclusion for online learners as they are perceived to be low priority, lack of readiness for online tools, difficulty communicating clearly and in real-time and feelings of isolation from the rest of the group and campus.
Toulouse, 2020, U.S.A	A: To observe how using SCRs for evaluation feedback might engage online students in their learning process. RD: Exploratory	*N* = 125 (*n* = 50 at a public university in 2017; *n* = 75 at a private university in 2019)	DS: Survey with open response questions	SCRs used to prove feedback on assignments enhance connectedness by conveying faculty tone, caring, and an authentic human presence to students, thereby improving the student learning experience.
Wingo et al., 2016, U.S.A	A: To examine the benefits and challenges of teaching nursing courses online. RD: Qualitative-correlational	*N* = 21 Online faculty (F), *n* = 9; Administrators (A), *n* = 6; instructional designers (ID), *n* = 6	DS: Interviews, course demonstrations, course documents	F report difficulty in meeting student communication demands; A and ID did not see it as an issue or thought that in fact, they were not available enough. F and ID expressed a significant need for individualized training. More clarity is needed from institution when communicating program objectives and policies regarding course design and delivery.
Xavier et al., 2020, Portugal	A: (1) To identify the social, family, academic, and behavioural changes related to the COVID-19 pandemic in nursing students, and (2) to characterize their perceptions of health, information, and compliance with the enhanced health and safety measures. RD: Descriptive-correlational (quantitative)	*N* = 425 mean age = 21.4 yrs	DS: Survey using the COVID-19 International Student Well-being Survey (*C19-ISWS*)	The change in teaching method and movement to e-learning was a significant stressor even without a significant change in workload and a decrease in hours spent on academics. Significant concern for their health lead to a strict compliance of COVID-19 enhanced Health and Safety measures. Of note, 64% of students reported decreased communication with friends. Regarding their health, there was a significant decrease in tobacco (*r* = 0.68), alcohol (*r* = 0.72), and cannabis (*r* = 0.76) consumption among users.
Yilmaz, 2019, Turkey	A: To determine the predictive power of Facebook adoption and virtual environment loneliness (VEL) on knowledge sharing behaviours (KSB). RD: Correlational	*N* = 279 Undergraduate students, Year 1	DS: Questionnaires TDC: Questionnaires administered after 1 semester	There is a moderate correlation between knowledge sharing behaviours and Facebook adoption (*p* < 0.01) and among KSB and VEL (*p* < 0.01). Feelings of low loneliness improved the KSB (*p* < 0.01). Overall, KSB was high when Facebook was adopted.
Zeren, 2015, Turkey	A: To investigate F2F and online, synchronous chat counselling in terms of client problems and satisfaction. RD: Qualitative	*N* = 21 mean age = 22.3 yrs	DS: Surveys and transcripts of counselling sessions TDC: Surveys administered at beginning and cessation; Transcripts from last session	Rationale for seeking counselling generally did not differ for both F2F and online clients. Findings suggest that F2F clients and online clients have similar rates of satisfaction.
Zhu & Van Winkel, 2016, Belgium	A: To investigate the extent to which a VLE supports the continuation of education and school interactions among sick adolescents and their well-being. RD: Mixed methods (qualitative & quantitative)	*N* = 28 mean age = 14.6 yrs	DS: Structured questionnaire; Interviews	Students reported being satisfied with both the academic and social benefits of the full VLE. Of significant interest was being able to keep formal and informal contacts and social interactions with classmates. The VLE played an important role in making social and academic connections possible, reducing the social stress of the students. The more the students were fulfilled with the use of the VLE in helping them continue their education, the higher the measurement of mental well-being (*p* < 0.01).

*Note: *3DVR: 3D Virtual Reality, 3DVE: 3D Virtual Environment, AR: Augmented Reality, BLM: Blended Learning Model, F2F: Face-to-Face, LMS: Learning Management System, SCR: Screen Capture Recording, VLE: Virtual Learning Environment

Our research provided studies that had been conducted from nearly every continent of Earth, save South America and Antarctica. Notably, many of the articles were completed by researchers in the United States (*n* = 16). Fifteen were completed by researchers in Europe (Belgium, Cyprus, Georgia, Germany, Greece, Poland, Portugal, Slovenia, The Netherlands, Turkey, Ukraine, and the United Kingdom), and six were completed by researchers in Asia (India, South Korea, Taiwan, and Vietnam). The remaining research was conducted in Australia, Canada, Jordan, and Kenya. The research designs employed by the various researchers in our summary are quite varied and sometimes particularly nuanced and range from case study to meta-analysis. However, a preference for experimental, quasi-experimental, qualitative analysis, and mixed-method correlational analysis did emerge. Overall, few articles had a singular focus, which can be attributed to the expansive field of VLEs and their many intricate pieces. Some central concepts were highlighted, however, in the research: fifteen observed the effectiveness, benefits or challenges of some aspect of VLE (i.e., using virtual reality simulations or synchronous video); ten investigated student readiness, either on a social-emotional basis, technology know-how, or academic; six focused on the mental health of the learners in VLEs; six gathered data on the experiences and perceptions of either students, students’ families, or faculty, and four researched the current infrastructure and available policies for online learning. The participant profiles of our research were also quite varied, identifying elementary and secondary school-aged children, both undergraduate and graduate students, school faculty, and student family members, sometimes all within the same study.

Our research results have been organized in Table 1 below. It has been constructed in such a manner whereby an informational narrative that reflects the essential themes found within the research can be revealed.

## Results and discussion of themes

The articles included in Table 1 represent the most current and relevant research in considering the embedded inquiry of this scoping review which involves uncovering the nature, implications, and best iterations of practice within VLE contexts. In our reading and review of the data therein, the themes of insufficient data surrounding VLEs, VLE benefits, the challenge of VLE readiness, and that which constitutes the ideal VLE emerged as pivotal. The objective of this section is to elucidate these themes, thereby, providing a modest basis for recommendations regarding VLE implementations and, perhaps, a view to offer directionality for future research.

### Insufficient data

A key note thread found within many of articles was the self-admission of insufficient data. This theme of insufficient data is expressed in varying capacities that range from claims of there being a limited or even non-existent body of research, to more systemic causes for the insufficiencies. While the lack of data is often presented as a cautionary device for the demarcation of limits to implementation outside the context of the studies and provide exhortation for further research to be conducted, the admissions of insufficient data also point to the novel nature of the area of inquiry in question. Kumar and Owston (2015) begin their study on e-learning accessibility by stating that their field of inquiry had “not been explored, nor have methods to generate data” (p. 264) expressing that there is “a dearth of studies'' (p. 268) in the literature, and concluding that “[c]ontinued work in the area of developing methods to evaluate e-learning accessibility is thus urgently needed” (p. 280). Archambault et al. (2013) also identified their research scope of basic virtual school policies as being novel in nature, having no representation in the existing literature. Many researchers make note of the existing data as being too insufficient to draw more universal conclusions (Barbour & LaBronte, 2019; Cavanaugh et al., 2004; Engelbertink et al., 2020; Gillis & Krull, 2020; Ho et al., 2014; Jena, 2016; Zhu & van Winkel, 2016). In addition to this paucity of research, the attrition of study participants is noted as being a barrier to gathering full data sets (Manthey et al., 2016).

Some systemic issues which led to shortages in the available data are noted in Johnston et al. (2014) where school districts are slow to institute policy. Cavanaugh et al. (2004) mentioned a similar dynamic in considering that common goals are needed in policy making to identify the effectiveness of an intervention and policy makers and evaluators are exhorted to work together in partnership to ameliorate this. A further systemic barrier to data production that is noted is the problem of implementation of programming without conducting research (Cavanaugh et al., 2004).

### Benefits of VLEs

In response to our first research question regarding the benefits of a wholly synchronous VLE experience, the research is generally favourable toward academic achievement with some degree of attestation to its social-emotional benefits. The benefits to VLEs and their implementation are assumed among most of our research in how they can be potential vehicles delivering some form of meaningful intervention or program within a given context. Further, some of the articles underline fundamental goods that can be uniquely exploited via VLEs. Driscoll et al. (2012) cites VLEs as an opportunity to better promote a constructivist framework for learning in saying that it inherently “creates a structural impetus for this style of learning that is not automatically present in F2F classrooms” (p. 314). Cavanaugh et al. (2004) provides multiple examples of how the institutional advantages of virtual schools “represent the best hope for bringing high school reform quickly to large numbers of students” (p. 22). Building upon the pervasive benefits to VLEs as a concept, Roblek et al. (2019) frame VLE dynamics as an essential component of human advancement where “social relations will be formed through the building of collective intelligence” (p. 96). Similarly, VLEs and their relation to ICT literacy as a global objective is observed throughout the research (Blayone et al., 2018; Cavanaugh et al., 2004; Crea & Sparnon, 2017; Davies, 2014; Gibson & Smith, 2018; Huang et al., 2011; Hursen, 2019; Jena, 2016; Mallya et al., 2019).

The strengths of specifically synchronous VLEs emerge in the research with highlighting synchronous learning as an essential component to student engagement with technology, peers, and educators. Concerning technology fluency, even in a blended learning context, synchronous VLEs offered a unique opportunity to implement technology in a meaningful way (Ho et al., 2016). Using a device in a synchronous context meant that students felt more engaged with material, subsequently feeling more confident with presenting work using technology, and students enjoyed being able to revisit an interactive lesson digitally after the synchronous session was over (Davies, 2014; Driscoll et al., 2012; Kumar & Owston, 2016). In terms of supporting engagement among classmates, synchronous learning was seen to offer increased avenues for peer-to-peer learning while allowing for teacher involvement throughout, thus increasing effectiveness (Crea & Sparnon, 2017; Johnston et al, 2014). Synchronous VLEs that include video also offer opportunities to be present to a class setting in a way that attends to learning retention, academic engagement, resiliency, and self-regulation (Archambault et al., 2013; Driscoll et al., 2012). When VLEs employ best-possible real-time communication, education processes can be more active, constructive, cooperative, and more attentive to a student’s meta-cognitive abilities than the traditional classroom (Cavanaugh et al., 2004). These latter points concerning real-time visual instruction potentially align with a foundational dynamic noted by Mesman et al. (2009) where it is stated that an “infant needs an external regulator to achieve optimal arousal levels and will show disorganization of emotion and behaviour when the regulator is absent or non-optimal” (p.122). Such a relationship becomes apparent in the work of.

Baker et al. (2019) which observed quiz results decrease among those students whose instructor withdrew communication and synchronous availability after originally being quite attendant to their needs and in the work of Engelbertink et al. (2020) where student motivation dropped significantly when the teacher no longer demonstrated an interest in the student’s homework. Throughout the research, it is evident that student engagement and achievement is well-supported in a synchronous VLE.

### The barrier to a VLE: the challenge of readiness

Across all our research, it became clear that one of the primary factors curtailing the effectiveness of any VLE or LMS was the various states of readiness of the institution, the teacher, and the student.

At an institutional level it can be said that most schools are not equipped to create VLEs where students can thrive, even those schools that are virtual by design. The infrastructure required to create a holistic learning experience for the student, and one that embodies fair and equitable working conditions for the online educator, requires a considerable investiture of human resources and technological tools (Archambault et al., 2013; Cairns et al., 2020; Jones, 2015). Many LMSs that institutions use for online learning are bulky and inefficient (Gillis & Krull, 2020; Jones, 2015; Kumar & Owston, 2016; Lee et al., 2016) which can lead to their being used as places where information is simply disseminated, rather than genuine VLEs where the design and curriculum content can come together to connect students with each other for interaction and collaboration (Jones, 2015; Stone, 2019). Elementary schools, for instance, can be said to provide many opportunities for families to increase their informal social capital and high schools, colleges and universities often provide a student with guidance and counseling services not easily accessible elsewhere. In moving to online learning, these institutions must not forget their “organizational brokerage” (Domina et al., 2021, p. 4) in facilitating and maintaining these social connections lest their students suffer in isolation (Crea & Sparnon, 2017). Ultimately, the VLE experience begins with the institution; if there is no commitment to ensuring the use of a high-quality LMS and no focus on securing and maintaining the human resource social supports that students and families have come to rely on the school to provide, then the mental health and academic achievement of its students can deteriorate (Cairns et al., 2020; Cavanaugh et al., 2004; Domina et al., 2021; Gillis & Krull, 2020; Jones, 2015; Lee & Oh, 2017; Merlin-Knoblich et al., 2019; Rogowska et al., 2020; Stone, 2019; Xavier et al., 2020; Zhu & van Winkel, 2016).

As Blayone et al. (2018) points out, vital to the VLE experience is “high quality activity design, strong environmental and motivational supports, and competent online facilitators” (p.15). Teacher readiness for both the technological scope of VLEs and for the new expectations that they are the sole social-emotional support for students and families (at the very least a proxy to such supports) is generally low. Training is essential for educators who are navigating new technologies and creating resources that provide meaningful opportunities for knowledge construction, reflection, and practice (Davies, 2014; Gibson & Smith, 2018). Teachers must also be taught how to “adjust and find their own rhythm, providing sufficient presence while avoiding feeling perpetually ‘on call’” (Jones, 2015, p. 227). Teachers lacked access to suitable training and felt ill-prepared to offer and provide to students with special needs or disabilities the appropriate accommodations within the VLE (Kent et al., 2018). Substantial professional development is needed to ensure that teachers know how to provide social opportunities in the VLE that encourages group work, formal and informal interactions, and peer-to-peer cooperative learning (Cavanaugh et al., 2004; Johnston et al., 2014; Zhu & Van Winkel, 2016). Cultivating this social-emotional component is an essential task of the online educator; when a student can trust their teacher and their classmates, their self-efficacy and motivation increases and generally so does their performance and progress (Johnston et al., 2014). To accomplish this, institutions must increase their efforts in training and supporting their faculty to be ready for online instruction (Crea & Sparnon, 2017).

Jena (2016) defines student learning readiness as “the body of skills needed by learners to learn” (p. 950). This body of skills and aptitudes includes, but is not limited to, motivation, self-regulation, perceived usefulness, confidence with using various technology, attitude, self-efficacy, computational abilities, communication skills, and research and critical thinking competence (Baker et al., 2019; Blayone et al., 2018; Du et al., 2019; Hursen, 2019; Johnston et al., 2014; Jones, 2015; Mallya et al., 2019). Beyond these attributes of learning readiness is also a strong necessity for a certain level of social-emotional maturity, most especially if the online learning was a result of the COVID-19 pandemic or of illness (i.e., not a free choice). Soft qualities such as resilience, flexibility, and positivity (Lee & Oh, 2017) made it more possible for students to survive the transition from the routine and collaboration of a physical classroom to the more solitary and independent learning space of the VLE (Crea & Sparnon, 2017; Gibson & Smith, 2018; Jena, 2016). In addition to these crucial factors, is the technology-readiness of students. Students may not have access to their own personal device to do their schoolwork, and if they do, there is no guarantee that it is a device equipped with the sufficient technological specifications to handle the resource heavy online tools or that the student has access to high-speed internet to allow full and equal participation in the lesson and VLE (Domina et al., 2021; Gillis & Krull, 2020; Hursen, 2019). It cannot be assumed that because students use technology at very high rates for personal relationships and entertainment that they can directly transfer those skills to the sophisticated and critical digital literacy necessary and conducive to learning in a VLE (Blayone et al., 2018; Roblek et al., 2019). Indeed, the various online tools that are familiar to institutions and educators are rarely in the purview of students, though when the need arises, students do want to be taught how to use the many programs and LMSs available to them effectively (Stone, 2019) and thus system readiness, student readiness, student inclusion, student achievement and teacher readiness are inseparable (Huang et al., 2011; Kumar & Owston, 2016; Pryjmachuk et al., 2012; Yilmaz, 2019).

### The ideal VLE

Among the reviewed articles, the answer to our second research question concerning the criteria of an ideal VLE emerged. VLEs which supported students both academically and emotionally and whereby online educators were engaged and motivated were highly organized and inventive, and if given that no barriers of readiness existed, could be implemented in every school system willing to pivot to this necessary focus. Firstly, policies and procedures that focus on the progress and social-emotional needs of the student must be in place (Archambault et al., 2013). This can only be achieved if a full set of human resources such as guidance teachers, attendance officers, counsellors and special education resource teachers are available both on a central campus and online (Johnston et al., 2014) offering “inclusion, communication, connection with others and proactive institutional support” (Stone, 2019, p. 7) by way of a school-home mentorship model (Barbour & LaBonte, 2019). In this way, the student’s isolation is lessened and, united with the educational team, the VLE teacher can focus on lending their subject and pedagogical expertise to their students (Driscoll et al., 2012; Du et al., 2019; Engelbertink et al., 2020; Wingo et al., 2016; Zhu & van Winkel, 2016). Secondly, the VLE must be easy to use, accessible, flexible, and innovative. Institutions must select uncomplicated LMSs for teachers to use to deliver their program. The expectations of use must also be communicated to all faculty to ensure a seamless experience for students (Jones, 2015). As well, in either a synchronous VLE or BLM, having easy access to recorded lessons is crucial, especially for students with disabilities or who are still learning the language (Davies, 2014; Dommett et al., 2019; Kumar & Owston, 2016). Investment in innovative tools and technologies is necessary to keep the VLE from becoming stagnant for students and, depending on the technology, can promote healthy, rich, and meaningful student interactions (Du et al., 2019). There is promising research in the use of tools such as AR, VR, 3DVR and 3DVE to create experiences and spaces that allow students to attend to one another virtually. These tools help to cultivate positive relationships, academic and personal confidence, and good mental health (Huang et al., 2019; Lan et al., 2018; Papanastasiou et al., 2019; Stone, 2019). Thirdly, there must be, at best, a live-video synchronous component to the VLE, or at minimum, the availability of synchronous office-hours (Stone, 2019; Wingo et al., 2016; Zeren, 2015; Zhu & van Winkel, 2016). When students and teachers were engaged face-to-face, body language and tone could be better understood and relationship markers such as trust and care could be better perceived (Driscoll et al., 2012; Johnston et al., 2014; Wingo et al., 2016). Finally, the VLE must engage students in becoming digital citizens together. VLEs that provide opportunities for students to engage formally and informally enable students to increase their academic self-efficacy, increase their learning outcomes, and mitigate any mental health issues that may result from the perceived isolation of online learning (Driscoll et al., 2012; Du et al., 2019; Engelbertink et al., 2020; Johnston et al., 2014; Stone, 2019; Yilmaz, 2019; Zhu & van Winkel, 2016).

### Discussion of gaps and limitations in the research and suggestions for further inquiry

The attempt to study any observable intersection of VLE implementation and student mental health presents unique logistical and philosophical queries that remain unquelled. Such wonderings involve the state of how participant numbers are determined, the founding modalities in which self-reported qualitative data is obtained, the rationale, or lack thereof, of why specific LMS platforms were used in the existing studies, and the generally perceived evolving nature of VLEs. Taken together, the various streams of inadequate information fret deeply and, perhaps, create quite significant gaps. In the following discussion of these gaps, we will humbly aim to make moderate suggestions for further inquiry that could enrich the current available research.

Concerning the limitations in obtaining meaningful participation, a key area that remained challenging among the research was ensuring that participant profiles were not assembled out of simply convenient contexts of implementation. Indeed, quality research is exhorted to communicate, as narrowly as possible, the contexts in which they are situated. However, our search yielded a number of studies that were isolated case studies (e.g., Archambault et al., 2013; Johnston et al., 2014; Jones, 2015; Kumar & Owston, 2016) or were relegated to being singularly quasi-experimental (e.g., Blayone et al., 2018; Davies, 2018; Driscoll et al., 2012; Ho et al., 2016; Huang et al., 2011; Lee et al., 2016) in nature due to the fact that their implementation was imposed upon pre-existing participant groupings – those who happened to be enrolled in the class that was chosen for intervention. In extension to this, adequate control conditions were not always apparent, especially those which considered many factors that were changed in the experience of intervention groups. That is all to say that the interventions themselves were multifaceted, and one could surmise a possible inability to distinguish which key facet or combination was pivotal in the intervention. This issue may be considered a specific function of the sheer complexity of studying VLE implementations themselves. It is further compounded in the noting of pre-existing intervention groupings as it is perhaps the result of simple pragmatism in observing VLE implementations where they are available to be observed. This point recognizes that VLEs require specific access to resources that may be limited, making widespread and universally approachable studies a challenge. Here, it is possible that an underlying dynamic exists in the research where actioning any opportunity for study, however limited, is better than conducting no study at all. In our view, further inquiry into VLE efficacy and its relation to the mental health of students, should endeavour to include randomized trials, whereby there is no observed previous relationship between the intervention group and the researcher.

Another limitation to this scoping review related to participant selection is the scale and size of many of the studies. Several studies combined the type of participant, blending the experiences of students, faculty, and education support staff, thus limiting a focus on the unique perspective of the student as the end-user (e.g., Crea & Sparnon, 2017; Dommett et al., 2019; Engelbertink et al., 2020; Johnston et al., 2014; Stone, 2019; Wingo et al., 2016). Additionally, some studies that reported findings concerning students directly were of an extremely small student sample size of thirty or less (e.g., Cairns et al., 2020; Dommett et al., 2019; Engebertink et al., 2020; Hursen, 2019; Johnston et al., 2014; Kumar & Owston, 2016; Lan et al., 2018; Wingo et al., 2016; Zeren, 2015; Zhu & van Winkel, 2016). We note this small sample size in order to frame the perceived usefulness of these studies in the Ontario education context noting that *Ontario Regulation* 484/20, s. 4(14.1) states that “the average size in a school year of a board's online learning classes shall not exceed 30”. It is our view that findings of studies with a less than thirty sample size should be interpreted cautiously, as the dynamics and pressures conspicuous in an average sized class cannot be accurately measured. For further inquiry, we would suggest research that included groups of whole divisions across multiple school boards allowing for parallel interpretation and consistency.

A further limitation of this scoping review is the lack of consistency in LMS research. In as many facets as teachers differ so too do the online VLE tools that may be utilized to deliver programming and the effects of each LMS’s nuances can be difficult to account for and isolate as non-contributing factors within the studies. Several studies looked specifically at the Blackboard LMS (e.g., Crea & Sparnon, 2017; Davies, 2014; Du et al., 2019; Engebertink et al., 2020; Kent et al., 2018; Lee et al., 2016; Pryjmachuk et al., 2012) noting that in most cases its use was pragmatically chosen as it was already in use by the hosting institution. As well, multiple studies looked at either outdated programs, such as Facebook and RSS feeds (e.g., Huang et al., 2011; Hursen, 2018; Yilmaz, 2019) or expensive and new technology, such as 3DVR and iPads (e.g., Davies, 2014; Huang et al., 2019; Lan et al., 2018), that would be quite financially out of reach for most Ontario school boards to implement in any widespread and equitable fashion. Overall, researchers instead focused on studying only the perceptions of online learning in general or one specific piece of the online learning experience (for instance, the posting of recorded lectures or an asynchronous discussion board section) without giving any precise attention to the LMS used to create the VLE. In fact, it can be seen that the largest gap in the research is ignoring the LMS as a true, unto itself environment. We find this to be crucial to our research focus of determining how the perceived humanity of the VLE affects the mental health of the student; after all, it is the space in which the student will be spending most of their learning hours.

Many of the studies relied upon qualitative data gathered via surveys during the intervention which required participants to self-report on their perceived well-being and mental health. This paradigm of understanding reads as akin to consumer-based research where one who is satisfied with a product is more likely to repeat consumption regardless of whether the product ultimately increases quality of life. Just as the terms pleasure and contentment are not interchangeable, in the context of the research, it remains unclear if a participant’s self-reporting on perceived levels of anxiety is congruent with clinical definitions of the terms. While studies which utilized this form of data collection are free of equivocation by way of maintaining qualifying language such as “perceived level of anxiety” and not simply state “anxiety”, the question of the results being meaningful still remains. A suggestion for further inquiry may entail the implementation of standardized data sources such as the *PSS-10*, the *CES-D*, and the *MBI-SS* utilized by Lee and Oh (2017). Caution must be employed when developing questionnaires, interview questions, and surveys that provide opportunities to participants for open-ended self-reporting.

A final point on the limits of VLE research rests in the concern that LMSs may evolve at rates that do not allow for consistent implementation and ample research to be conducted in a timely manner. In an earlier section of this scoping review, we referenced the lack of research as a product of the field being relatively novel in nature. However, for several years now, LMSs have offered functionalities that extend into the realm of real-time collaboration that is inclusive of visual presence with teachers as well as being fully capable of allowing students fluid peer-to-peer real-time engagement. In considering this it is astonishing that our research showed such a vast range in how VLE operated in relation to the available technology. This has led us to wonder if the technology is available, why is it not being utilized and studied in a way that reflects its full capabilities? Aside from the concepts of readiness that have been earlier itemized and discussed, the level of investment that an institution is willing to make of a platform and the rate of making that LMS available with a fully optioned suite, becomes the pivoting element. Further, if a gradual investment model is employed, where not all features of the LMS are available at the onset, each time a new feature is doled out, it becomes a potential point of relearning and creates inefficiencies if there are not ample professional development opportunities for educators.

While determining our final research terms for this scoping review, we had initially searched for studies that were exclusively for the K-12 sphere and unsuspectingly this did not prove to be fruitful. We noted earlier that a peculiar number of studies had been published using nursing students as the study participants, reporting on their role as student and practitioner. We believe that the near exclusivity of undergraduate students in the research limited our ability to present a complete picture of the current state of VLEs for all students. In *The Human Face of Mental Health and Mental Illness in Canada*, the Government of Canada (2006) reported that “two-thirds (68.8%) of young adults aged 15–24 years with a mood or anxiety disorder reported that their symptoms had started before the age of 15” (p. 34). Boak et al. (2016) support these conclusions, showing in their report that “one-third (34%) of students indicate a moderate-to-serious level of psychological distress (symptoms of anxiety and depression)” (p. iv) and “one-in-eight (12%) students had serious thoughts about suicide in the past year” (p. iv), a statistic that has remained consistent over the past fifteen years of reporting. Given these unsettling statistics, we would have thought that the rich and varied K-12 arena would have been a sphere where there would be a surfeit of mental health research related to VLE utilization. Instead, our search results yielded studies that disproportionately represented participant groups outside of our desired range of inquiry; just six included the experiences of K-12 students, and in each of those studies, the students’ caregivers were the primary data source. K-12 students often spend more than half their waking hours in school environments and VLEs; it is notably unclear why most of the studies observed in this scoping review are unilaterally disinterested in exploring an identified area of need for mental health support. We believe it would be prudent to prioritize research on the mental health of K-12 students engaged in VLEs which Domina et al. (2021) has shown can be isolating, psychologically disruptive, and exhausting experiences.

## Suggestions for educator practice

Though there is variety in the identified gaps detailed above, our research maintained a consistent thread that related to the criteria of the ideal VLE for both the success of the educator but also the well-being and dignity of the student. From this work, we endeavour to make a few moderate suggestions for online educators:

- Where possible and where privacy concerns can be mitigated, conduct lessons and office hours using live videoconferencing. Whether the VLE is a secondary component in a BLM arrangement, or it is the primary mode of program delivery, maintaining a personal face-to-face connection is an essential component to a student’s feelings of connectedness and motivation.
- Avoid viewing the VLE as merely a space to bank work packages and collect evaluations. Rather, aim to create a space where both formal and informal interactions can occur, synchronously and asynchronously. Many LMSs have the capability to incorporate a variety of third-party online teaching and learning tools to aid educators in creating a multi-modal experience for students.
- Be vulnerable and take advantage of learning opportunities when they become available. It takes an enormous amount of energy and resources to run stimulating programs that speak honestly to curriculum content, allow for individual learning needs, and that are cognizant to the social-emotional well-being of students. The responsibilities and conditions of an online educator are well-primed for strain; be mindful of the added pressure and allow the professional development that is available to inform practice but not make hurried demands of that practice.

## Conclusion

If educational research involves an ethical component, it would be incumbent on institutions to see that research reflects areas of need within communities. It is our hope that this scoping review might provide modest insight into the current state of research that concerns student mental health in VLE contexts, while casting light on the need for new research initiatives to be undertaken in the K-12 sphere. As it stands, there lies the strong possibility that K-12 students are experiencing VLE implementations that do not actively partake in the qualities of a VLE that soundly offer best practices, working to support the mental health needs of students. To build strong VLE’s for K-12 students, research campaigns ought to offer architectures that are universalized in their implementation and fundamentally repeatable. This requires a commitment beyond that of the researchers involved, but also a willingness of the institutions who serve the participants of such a sweeping study to abide by the research. Without such research, institutions which utilize VLEs can only continue on sometimes arbitrary perceptions of how best to serve student wellness. Persisting in the status quo as such leaves students vulnerable to practices that might institutionally under-serve them and have potential generational implications. Interestingly, one might argue that, without such research, institutions who offer VLEs might garner the ability to omit themselves of the direct responsibility to provide those qualities of VLEs that would be found to support mental health and exclude those qualities that are found to diminish mental health.

As a closing thought and to return to the experiential modus and inquiry of this review, we adjure future research to be guided by the question of how the student encounters their teacher within the VLE. Emmanuel Levinas, a philosopher who wrote extensively on the innate ethical experience that is garnered through face-to-face interaction, took a rare moment in his writing to offer insight on the dynamics of education. In Levinas’ *Totality and Infinity,* he notes that in being called to respond to the Other, “[teaching] designates an interior being that is capable of a relation with the exterior and does not take its own interiority for the totality of being” (Levinas, 1969, as cited in Zhao, 2016, p. 324). Here, Levinas may appear to point to the disposition of the educator as one that offers the presence of self for the sake of the students’ being. This sentiment, taken along with the intriguing meta-analysis offered by Mesman et al. (2009) may do little to establish the “how” of education as conveyed through this inquiry, but certainly makes a tremendous stride in the realm of the “why” that institutions ought to work to expound among the current VLE modalities that they are imposing upon learning communities.

## Data Availability

Not Applicable.
